# Implications of AB0 blood group in hypertensive patients with covid-19

**DOI:** 10.1186/s12872-020-01658-z

**Published:** 2020-08-14

**Authors:** Celestino Sardu, Raffaele Marfella, Paolo Maggi, Vincenzo Messina, Paolo Cirillo, Vinicio Codella, Jessica Gambardella, Antonio Sardu, Gianluca Gatta, Gaetano Santulli, Giuseppe Paolisso

**Affiliations:** 1 Department of Advanced Medical and Surgical Sciences, University of Campania “Luigi Vanvitelli”, Naples, Italy; 2 Department of Medical Sciences, International University of Health and Medical Sciences “Saint Camillus”, Rome, Italy; 3 Department of Mental Health and Physics and Preventive Medicine, University of Campania “Luigi Vanvitelli”, Caserta, Italy; 4 Department of Advanced Biomedical Sciences, International Translational Research and Medical Education Academic Research Unit (ITME), “Federico II” University, Naples, Italy; 5 Department of Medicine and Department of Molecular Pharmacology, Wilf Family Cardiovascular Research Institute, Albert Einstein College of Medicine - Montefiore University Hospital, New York, NY USA; 6 Federazione Italiana di Medicina Generale, FIMG, Naples, Italy

**Keywords:** Covid-19, Hypertension, Coagulopathy

## Abstract

**Background:**

Hypertension is the most frequent co-morbidity in patients with covid-19 infection, and we might speculate that a specific blood group could play a key role in the clinical outcome of hypertensive patients with covid-19.

**Methods:**

In this prospective study, we compared 0 vs. non-0 blood group in hypertensive patients with covid-19 infection. In these patients, we evaluated inflammatory and thrombotic status, cardiac injury, and death events.

**Results:**

Patients in non-0 (*n* = 92) vs. 0 blood group (*n* = 72) had significantly different values of activated pro-thrombin time, D-dimer, and thrombotic indexes as Von Willebrand factor and Factor VIII (*p* < 0.05). Furthermore, patients in non-0 vs. 0 blood group had higher rate of cardiac injury (10 (13.9%) vs. 27 (29.3%)) and death, (6 (8.3%) vs. 18 (19.6%)), (*p* < 0.05). At the multivariate analysis, Interleukin-6 (1.118, CI 95% 1.067–1.171) and non-0 blood group (2.574, CI 95% 1.207–5.490) were independent predictors of cardiac injury in hypertensive patients with covid-19. D-dimer (1.082, CI 95% 1.027–1.140), Interleukin-6 (1.216, CI 95% 1.082–1.367) and non-0 blood group (3.706, CI 95% 1.223–11.235) were independent predictors of deaths events in hypertensive patients with covid-19.

**Conclusions:**

Taken together, our data indicate that non-0 covid-19 hypertensive patients have significantly higher values of pro-thrombotic indexes, as well as higher rate of cardiac injury and deaths compared to 0 patients. Moreover, AB0 blood type influences worse prognosis in hypertensive patients with covid-19 infection.

## Background

Hypertension is the most common co-morbidity and cause of death in patients with covid-19 infection [1]. Such a negative correlation between hypertension and clinical prognosis in covid-19 patients has been deeply investigated in recent trials [1, 2]. Angiotensin converting enzyme 2 (ACE2), known to be involved in the molecular pathways underlying hypertension, is a crucial co-factor mediating SARS-CoV-2 entry into host cells [2]. Indeed, the spike proteins of SARS-CoV-2 have a high binding affinity for ACE2, which are mainly expressed in endothelial cells of the lung and the upper airways [3]. Moreover, angiotensin converting enzyme inhibitors (ACEi) and angiotensin receptor blockers (ARB) have been shown to up-regulate ACE2 levels, which partially mediates their cardiovascular protective effects [4]. Nevertheless, according to the recent evidence, ACEi/ARB therapy does not seem to increase the risk of covid-19 infection in hypertensive patients [4]. Secondly, ACEi/ARB therapy discontinuation is not recommended, because it may lead to endothelial dysfunction [4]. Actually, endothelial dysfunction itself, mirrored by hyper-inflammation could cause alterations of the coagulation thereby aggravating the prognosis of the disease [2, 3].

The AB0 blood group has been previously shown to play a functional role in viral infections [5, 6]. Intriguingly, patients with non-0 blood group have higher risk for covid-19 infection when compared to 0 blood groups [7], and the AB0 blood group could influence the coagulation processes [8, 9]. However, the pathogenic mechanisms underlying these events have not been fully investigated, and could be of great interest to the scientific community and for clinical applications.

Thus, we hypothesized that non-0 blood group could be a trigger of endothelial dysfunction, via over-inflammation and promoting a pro-thrombotic status in hypertensive patients with covid-19. Actually, although hypertension is known to trigger endothelial dysfunction and a pro-thrombotic status [9], no data are currently available exploring the association of AB0 group with inflammatory/thrombotic status in hypertensive patients with diagnosis of covid-19.Therefore, in this study we investigated the inflammatory/thrombotic status and clinical outcomes as cardiac injury and death in hypertensive patients with covid-19,comparing 0 vs. non-0 blood groups.

## Methods

In this prospective study, we analyzed covid-19 hypertensive patients consecutively admitted to the Department of Infectious Disease at University of Campania “Luigi Vanvitelli”, Naples, Italy between February 10, 2020 and April 20, 2020. Covid-19 infection was categorized as follow: a) Mild, patients with fever and no pneumonia evidence in imaging; b) Moderate, patients with fever, respiratory tract symptoms, pneumonia confirmed at imaging without the need for invasive ventilation; c) Critical, occurrence of respiratory failure requiring mechanical ventilation, presence of shock, other organ failure requiring monitoring and treatment in intensive care unit [1].

### Exclusion criteria

patients with previous inflammatory disorders, malignancy, renal diseases; unavailability of a written informed consent; patients without cardiac biomarkers evaluation, including values of high-sensitivity troponin I (hs-TNI) and creatinine kinase–myocardial band (CK-MB). The diagnosis of hypertension was made following the international guidelines [10], and/or by known history of hypertension and current anti-hypertensive therapy.

All enrolled patients were treated with the same standard protocol: non-invasive oxygen therapy; hydroxychloroquine (400 mg/daily) and lopinavir/ritonavir (200/50 mg daily). According to AB0 blood group, patients were then categorized as “0 and non-0 blood group”, [6, 7]. Established cardiac biomarkers, including hs-TNI, CK-MB, and myo-hemoglobin, were collected for every participant at hospital admission by 2 investigators (V.M. and C.S.).The investigation conforms to the principles outlined in the Declaration of Helsinki for the study of human subjects or tissues. The institutional ethics committee of the University of Campania “Luigi Vanvitelli” approved the study protocol. Written informed consent was obtained from all participating patients.

### Study outcomes

In this study, we investigated the inflammatory and coagulative status, and the cardiac injury and deaths in hypertensive patients with covid-19, with the aim to compare 0 vs. non-0 blood group. Cardiac injury and death were reported in a previous study for patients with covid-19 [11]. Cardiac injury was defined as blood levels of cardiac biomarkers (hs-TNI) above the 99th-percentile upper reference limit (11). Data on cardiac injury and death were collected by two independent physicians (P.M; R.M) during clinical examination, laboratory and imaging tests in hospitalized patients, and by examination of hospital discharge schedules [11].

### Laboratory and imaging evaluations

#### -real-time reverse transcription (RT-PCR assay for SARS-CoV-2

Respiratory specimens were collected from each patient and then shipped to specialized laboratories designated by the Italian government for confirming covid-19 infection. The presence of SARS-CoV-2 in respiratory specimens was detected by established RT-PCR methods. Laboratory analyses were obtained on admission before starting covid-19 medical therapy and during hospitalization.

#### -Clinical and laboratory parameters

We tested respiratory specimens, including nasal and pharyngeal swabs or sputum, to exclude evidence of other viral infections, including influenza, respiratory syncytial virus, avian influenza, para-influenza, and adenovirus. We also performed routine bacterial and fungal examinations. Laboratory assessments consisted of a complete blood count, blood chemical analysis, coagulation testing, evaluation of liver and renal function, and measures of electrolytes, C-reactive protein, procalcitonin, lactate dehydrogenase, and creatine kinase. Venous blood for IL-6 (Human ELISA Kit, RD System) and D-dimer (Human ELISA Kit, Invitrogen) levels was collected in EDTA-coated tubes immediately after patients arrived at the department and weekly during hospitalization.

The AB0 phenotypes were ascertained by genotyping for four single nucleotide polymorphisms of the *AB0* gene: G261del, A297 G, G703A and C526G, as described [9]. Briefly, we used single nucleotide polymorphisms of the C526G to decipher the O303 allele, which, unlike other O alleles, does not have a deletion at nucleotide position 261 [9]. We determined genotyping by using the multiplexing capability of the MassARRAY homogenous MassEXTEND assay of the Sequenom system (San Diego, CA, USA). Therefore, the DNA fragments surrounding the single nucleotide polymorphisms sites were amplified by PCR, treated with shrimp alkaline phosphatase to dephosphorylate unincorporated dNTPs, followed by the extension primers that form allele-specific extension products. However, each extension product had a unique mass, measured using MALDI-TOF. Genotypes were automatically assigned to each sample using the Mass ARRAY RT software. The presence or absence of FV Leiden (A1691 G, R506Q) and the prothrombin G20210A polymorphism was assessed by standard methods [9]. All patients underwent ECG at hospital admission, and in case of elevation of cardiac biomarkers during hospitalization; findings compatible with myocardial ischemia included T-wave depression and inversion, ST-segment depression, and Q waves. Two blinded physician (C.S, R.M) reviewed and analyzed ECG patterns. Radiologic assessments included chest radiography and/or computed tomography (CT) at admission and weekly during hospitalization, and all laboratory testing was performed according to the clinical care needs of each patient. We determined the presence of radiologic abnormalities on the basis of the documentation or description in medical charts; if imaging scans were available, they were reviewed by attending physicians in respiratory medicine who extracted the data. Two blinded physician experienced in lung imaging (G.G, V.C.) reviewed and analyzed chest radiography and CT patterns. Major disagreement between two reviewers was resolved by consultation with a third reviewer.

### Statistical analysis

Continuous variables were expressed as medians and interquartile ranges or simple ranges, as appropriate. Categorical variables were summarized as counts and percentages. We performed only descriptive statistics, because the cohort of patients in our study was not derived from random selection. We performed a risk adjusted Cox-regression analysis to assess survival from cardiac injury and deaths through days of hospitalization; Cox models were adjusted for; age, gender, body mass index, heart rate, cholesterol, high density lipoprotein-cholesterol, low density lipoprotein-cholesterol, triglycerides levels, heart diseases, dyslipidemia, diabetes, current smoking, beta-blockers, ace-inhibitors, calcium inhibitors, thiazide diuretics, aspirin. Only variables presenting a *p* value ≤0.25 at the univariate analysis were included in the model. We used a stepwise method with backward elimination, and we calculated odds ratios (OR) with 95% confidence intervals. The model was evaluated with a Hosmer and Lemeshow test. Kaplan-Meier survival analysis was performed for cardiac injury events and deaths in patients divided in: 0 vs. non-0 blood group. A *p* value < 0.05 was considered statistically significant. All calculations were performed using the software SPSS23.

## Results

We enrolled 164 hypertensive COVID-19 patients; the study population was then divided according to the AB0 blood group in0 (*n* = 72) vs. non-0 (*n* = 92). The main clinical characteristics of our population are shown in Table 1. Comparing 0 vs. non-0 blood group, we found significantly different values of activated pro-thrombin time, D-dimer, and thrombotic indexes including activated pro-thrombin time, Von Willebrand factor (VWF) and Factor VIII (*p* < 0.05).

**Table 1:** Clinical characteristics of study population

Clinical study variables	Overall (n164)	Group 0 (n72)	Group non-0 (n 92)	***P*** value
Age (years)	55 ± 18	52 ± 15	54 ± 19	0.232
Sex (male, %)	108 (65.8)	44 (61.1)	64 (69.5)	0.499
Smoking (%)	18 (10.9)	8 (11.1)	10 (10.9)	0.183
Body mass index (kg/m^2^)	25.5 ± 6.6	24.8 ± 7.3	26.3 ± 5.5	0.159
**Signs and symptoms at admission**				
Fever	131 (79.9)	57 (79.2)	74 (80.4)	0.076
Cough	57 (34.7)	24 (33.3)	33 (35.9)	0.376
Shortness of breath	47 (28.6)	21 (29.2)	26 (28.2)	0.560
Fatigue	31 (18.9)	14 (19.4)	17 (18.4)	0.560
Sputum production	8 (4.9)	3 (4.2)	5 (5.4)	0.502
Muscle ache	10 (6.1)	4 (5.5)	6 (6.5)	0.533
Diarrhea	8 (4.8)	3 (4.2)	5 (5.4)	0.502
Chest pain	11 (6.7)	5 (6.9)	6 (6.5)	0.233
Sore throat	8 (4.8)	4 (5.5)	4 (4.3)	0.498
Rhinorrea	8 (4.8)	3 (4.2)	5 (5.4)	0.502
Headache	8 (4.8)	4 (5.5)	4 (4.3)	0.498
**Chronic medical illness**				
Diabetes (%)	42 (25.6)	18 (25)	24 (26.1)	0.443
Coronary heart disease (%)	56 (34.1)	26 (36.1)	30 (32.6)	0.522
Previous AMI	30 (18.3)	13 (18.0)	17 (18.5)	0.156
CABG	8 (4.8)	4 (5.5)	4 (4.3)	0.498
PTCA	47 (28.6)	21 (29.2)	26 (28.2)	0.560
Chronic obstructive pulmonary disease(%)	26 (15.8)	11(15.3)	15 (16.3)	0.295
Cerebrovascular disease (%)	18 (11.0)	7 (9.7)	11 (11.9)	0.232
Chronic renal failure (%)	16 (9.7)	8 (11.1)	8 (8.7)	0.185
Cancer	13 (8)	5 (6.9)	8 (8.7)	0.498
**Laboratory findings at admission**				
Red blood cells, n ×10^6^ (μ/L)	3.8 [3.6–4.4]	3.8 [3.7–4.0]	3.9 [3.6–4.1]	0.785
Hemoglobin, g/dl	12.1 [10.8–13.9]	12 [11.5–13.4]	12.2 [11.7–13.3]	0.087
Whyte blood cells, n (μ/L)	8050 [3810–11,340]	7973 [3496–10,389]	8263 [3727–10,593]	0.122
Lymphocytes, n (μ/L)	974 [568–1128]	983 [672–1347]	978 [589–1132]	0.101
Neutrophils, n (μ/L)	6938 [2410–10,198]	6875 [1852–7899]	6943 [1972–8101]	0.226
Pro-thrombin time (PT), s	12.7 [12.1–15.3]	12.6 [12.1–15.2]	12.9 [12.1–15.8]	0.064
APTT (s)	29.3 [27.5–35.6]	28.5 [27.8–32.2]	31.1 [20.1–32.1]	0.002*
D-dimer (mg/mL)	2.68 [0.11–24.45]	1.62 [0.11–20.21]	3.8 [0.14–24.45]	0.009*
Von Willebrand factor (%)	239 [115–476]	209 [115–401]	256 [115–476]	0.007*
Factor VIII (%)	188 [115–355]	166 [115–336]	188 [115–356]	0.004*
Cholesterol, mg/dl	157.4 ± 14.7	157.5 ± 14	156.5 ± 15	0.953
AST (Aspartate aminotransferase), mg/dl	43 ± 32	45 ± 33	39 ± 32	0.137
ALT (Alanine amino transferase), md/dl	45.5 ± 27	47 ± 28	43 ± 24	0.131
CK-MB (Creatinine kinase-myocardial band), mg/dl	150 ± 16	149 ± 17	150 ± 19	0.984
LDH, mg/dl	608 ± 146	618 ± 14	596 ± 20	0.380
High sensitivity Troponin I, μg/L	0.39 [0.12–1.47]	0.38 [0.12–1.49]	0.40 [0.13–1.57]	0.943
Myohemoglobin, μg/L	49.92 ± 28.3	49.86 ± 30.1	49.46 ± 33.7	0.930
Creatinine, mg/dL	0.90 ± 0.22	0.92 ± 0.18	0.88 ± 0.25	0.118
BNP, pg/ml	35.5 ± 3.1	36.8 ± 3.7	31.4 ± 2.9	0.132
Glucose, mg/dl	131 ± 38	130 ± 37	132 ± 39	0.994
Hb1Ac, %	5.8 ± 0.4	5.8 ± 0.6	5.7 ± 0.9	0.654
Sodium, mEq/L	135.6 ± 2.6	135.6 ± 2.4	135.4 ± 2.5	0.303
Potassium, mEq/L	3.7 ± 0.2	3.6 ± 0.2	3.7 ± 0.3	0.703
PaO_2_/FiO_2_, mmHg	81 [64–109]	78 [66–108]	82 [72–110]	0.354
**Inflammatory markers**				
Interleukin 1, pg/dl	387.5 [321.8–422.1]	383.4 [332.6–404.5]	389.9 [339.8–408.9]	0.804
Interleukin 6, pg/dl	243.2 [202.7–251.2]	242.1 [216.8–248.9]	245.3 [222.1–250.1]	0.989
Tumor necrosis alpha, mg/dl	3.1 [1.94–4.89]	2.9 [2.6–4.32]	3.3 [3.0–4.64]	0.693
hs-C Reactive Protein, mg/dl	6.2 [1.2–17.12]	5.7 [4.3–16.7]	5.6 [1.2–18.7]	0.472
Procalcitonin, ng/ml	0.21 [0.04–0.44]	0.22 [0.06–0.39]	0.24 [0.05–0.46]	0.372
**Echocardiographic parameters**				
LVTDd, mm	46.9 ± 4.4	46.5 ± 4.5	47.2 ± 4.3	0.329
LVTSd, mm	31.1 ± 2.6	31.4 ± 2.8	30.7 ± 2.3	0.058
LVEF (Left ventricle ejection fraction), %	51.3 ± 6.7	51.6 ± 7.8	50.7 ± 5.6	0.461
Mitral insufficiency:				
Low (%)	109 (66.4)	45 (62.5)	64 (69.5)	0.436
Moderate (%)	55 (33.5)	25 (34.7)	30 (32.6)	0.483
Severe (%)	/	/	/	/
**Chest radiography and computed tomography findings**				
Pneumonia:				
Unilateral	39 (23.8)	18 (25)	21 (22.8)	0.747
Bilateral	124 (75.6)	54 (75)	70 (76.1)	0.907
Multiple motting and ground-glass opacity	87 (53)	38 (52.8)	49 (53.2)	0.843
**Chronic drug therapy**				
Anti-platelets(%):				
Cardioaspirin	42 (25.6);	20 (27.8)	22 (23.9)	0.067
Clopidrogel	39 (23.8);	18 (25)	21 (22.8)	0.747
Beta blockers, (%)	55 (33.5)	22 (30.5)	33 (35.9)	0.145
Angiotensin Converting Enzyme inhibitors, (%)	41 (25)	20 (27.8)	21 (22.8)	0.292
Angiotensin receptor blockers (%)	42 (25.6)	18 (25)	24 (26.1)	0.510
Calcium blockers (%)	18 (10.9)	8 (11.1)	10 (10.9)	0.510
Loop diuretics (%)	18 (11.0)	7 (9.7)	11 (11.9)	0.232
Thiazides(%)	31 (18.9)	14 (19.4)	17 (18.4)	0.560
Statins (%)	57 (34.7)	22 (30.6)	35 (38)	0.202
Hypoglycemic drugs (%)	20 (12.2)	6 (8.3)	14 (15.2)	0.445
Insulin therapy (%)	10 (6.1)	3 (4.2)	7 (7.6)	0.283
**COVID-19 therapy**				
Antiviral (%)	164 (100)	72 (100)	92 (100)	/
Antibiotics (%)	140 (85.4)	63 (87.5)	77 (83.7)	0.396
Chinidine (%)	134 (81.7)	60 (83.3)	74 (80.4)	0.395
Glucocorticoids (%)	128 (78)	57 (79.2)	71 (77.2)	0.522
Tocilizumab (%)	18 (10.9)	8 (11.1)	10 (10.9)	0.510
Oxygen inhalation (%)	132 (80.5)	57 (79.2)	75 (81.5)	0.271
Non-invasive ventilation (%)	34 (20.7)	15 (20.8)	19 (20.6)	0.540
**Study endpoints**				
Hospital admissions at Intensive Care Unit (%)	32 (19.5)	13 (18)	19 (20.6)	0.330
Mechanical Ventilation (%)	69 (42.1)	30 (41.7)	39 (42.4)	0.471
Cardiac injury (%)	37 (22.6)	10 (13.9)	27 (29.3)	0.014*
Death (%)	24 (14.6)	6(8.3)	18(19.6)	0.034*

Characteristics of the study population, and of 0 vs. non-0 group of patients. Categorical variables are shown as frequency rates and percentages, and continuous variables as mean (SD) and median (interquartile range [IQR]) for laboratory findings at admission. We indicated High sensitivity Troponin I (μg/L) as median (interquartile range [IQR]), and Myohemoglobin (μg/L), AST (mg/dl), ALT (mg/dl), CK-MB (mg/dl) and LDH (mg/dl) as means ± standard deviations. The means for continuous variables were compared using independent group t tests when the data were normally distributed (normal distribution verified applying the Kolmogrov-Smirnov test), otherwise, the Mann- Whitney test was used. The Pearson correlation coefficient and Spearman rank correlation coefficient were used for liner correlation analysis. Proportions for categorical variables were compared using the χ^2^ test, whereas the Fisher exact test was used when data were limited. Wilcoxon rank sum matched- pair tests were used to assess differences among the admission, hospitalization, and impending death. A 2-sided *P* < .05 was considered statistically significant. Analysis began February 29, 2020

*AMI* acute myocardial infarction, *CABG* coronary artery bypass grafting, *PTCA* percutaneous coronary angioplasty, *PT* Pro-thrombin time, *APTT* activated pro-thrombin time, *AST* aspartate amino transferase, *ALT* alanine amino transferase, *CK-MB* Creatinine kinase-myocardial band, *LDH* lactate dehydrogenase, *BNP* B type natriuretic peptide, *Hb1Ac* glycated hemoglobin, *PaO2/FiO2* Pressure of Arterial Oxygen to Fractional Inspired Oxygen Concentration, *hs* high specificity, *LVTDd* left ventricle end-diastolic diameter, *LVTSd* left ventricle end-systolic diameter, *LVEF* left ventricle ejection fraction, * is for statistical significant (*p* < 0.05)

Patients in non-0 vs. 0 blood group had higher rate of Cardiac injury [10 (13.9%) vs. 27 (29.3%)] and Deaths [6 (8.3%) vs. 18 (19.6%)], (p < 0.05) as shown in Table 1. Cardiac injury was diagnosed in 13 (54%) of patients.

Then, we performed a multivariate analysis, which revealed that interleukin-6 (IL-6, 1.118, CI 95% 1.067–1.171) and non-0 blood group (2.574, CI 95% 1.207–5.490) were identified as independent predictors of cardiac injury in hypertensive patients with covid-19 (Table 2).

**Table 2:** The multivariate study of the prognostic influence of various parameters on cardiac Injury events

	HR	Univariate Analysis CI 95%	***P*** value	HR	Multivariate Analysis CI 95%	***P*** value
ARB	0.998	0.471–2.114	0.995	0.980	0.436–2.203	0.962
Aspirin	0.749	0.370–1.516	0.422	0.529	0.244–1.148	0.107
BMI	1.002	0.997–1.008	0.397	1.007	0.997–1.017	0.201
Diabetes	0.388	0.201–1.748	0.065	0.774	0.307–1.954	0.588
D-dimer	1.053	1.013–1.095	0.009	0.996	0.951–1.044	0.874
Group non-0	2.212	1.070–4.571	0.032	2.574	1.207–5.490	0.014*
IL-6	1.114	1.067–1.163	0.001	1.118	1.067–1.171	0.001*
Sex	2.623	1.373–5.012	0.004	2.343	1.096–5.009	0.028
hs-Troponin I	0.371	0.119–1.150	0.086	0.477	1.141–1.606	0.232
WBC	1.001	0.884–1.051	0.419	1.005	0.889–1.101	0.867

In this table the results of multivariate analysis for prognostic influence of various parameters on cardiac injury study endpoint, done by Cox regression analysis with confidence interval (CI) 95%. *ARB* Angiotensin Receptor blockers, *BMI* Body mass index, *HR* Hazard ratio, *hs* high sensitivity, *IL-6* Interleukin 6, *WBC* White blood cells; *:*p* < 0.05

Moreover, at multivariate analysis, D-dimer (1.082, CI 95% 1.027–1.140), IL-6 (1.216, CI 95% 1.082–1.367) and non-0 group (3.706, CI 95% 1.223–11.235) were identified as independent predictors of death in hypertensive patients with covid-19 (Table 3).

**Table 3:** The multivariate study of the prognostic influence of various parameters on deaths events

	HR	Univariate Analysis CI 95%	P value	HR	Multivariate Analysis CI 95%	P value
ARB	0.987	0.392–2.485	0.977	0.220	0.431–3.456	0.708
Aspirin	0.680	0.291–1.590	0.374	0.484	0.187–1.254	0.135
BMI	1.003	0.996–1.010	0.469	1.008	0.986–1.014	0.975
Diabetes	1.397	0.176–1.894	0.086	1.446	0.131–1.161	0.091
D-dimer	1.095	1.051–1.140	0.001	1.082	1.027–1.140	0.003*
Group non-0	2.446	0.971–6.164	0.058	3.706	1.223–11.235	0.021*
IL-6	1.213	1.080–1.362	0.001	1.216	1.082–1.367	0.001*
Sex	1.757	0.787–3.922	0.169	0.779	0.318–1.908	0.585
hs-Troponin I	1.190	0.478–2.963	0.708	1.446	0.443–4.722	0.541
WBC	0.893	0.135–1.045	0.126	1.012	0.872–1.101	0.583

In this table the results of multivariate analysis for prognostic influence of various parameters on deaths study endpoint, done by Cox regression analysis with confidence interval (CI) 95%. *ARB* Angiotensin Receptor blockers, *BMI* Body mass index, *HR* Hazard ratio, *hs* high sensitivity, *IL-6* Interleukin 6, *WBC* White blood cells; *:*p* < 0.05

**Fig. 1:**
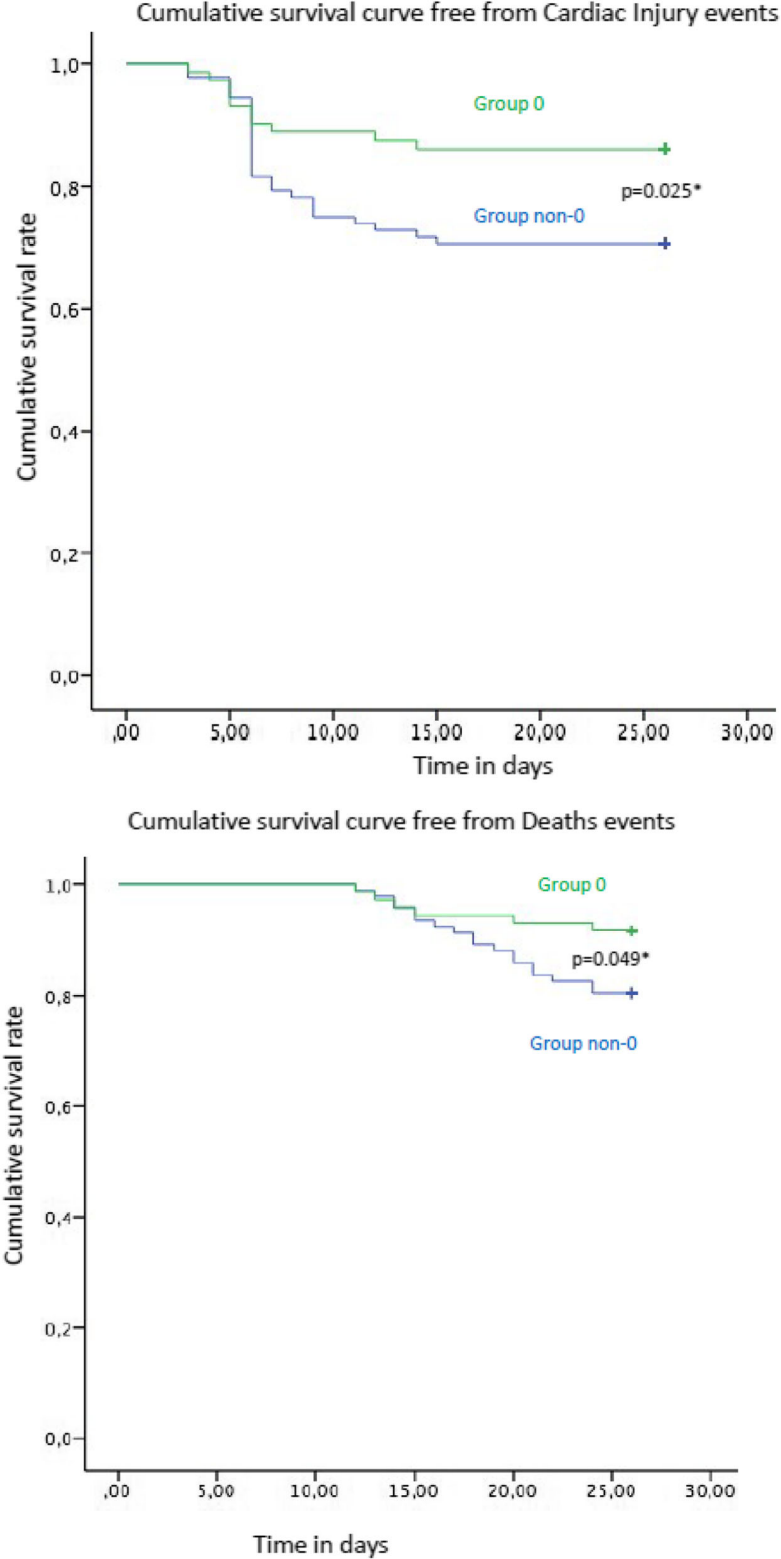
In this figure the actuarial probabilities calculated according to Kaplan-Meier survivor curve free from Cardiac Injury (upper part, χ 2 = 5.045, *p* = 0.025), and for Deaths (lower part, χ 2 = 3.880, p = 0.025). Green color: group 0; blu color: group non-0; *:*p* < 0.05

Finally, we analyzed Kaplan curves of survival (Fig. 1), observing a significant (*p* < 0.05) difference between O vs. non-0 hypertensive patients with covid-19 in terms of cardiac injury (upper panel) and death (lower panel).

## Discussion

From the analysis of hypertensive patients with covid-19 infection, the main study results are: i) non-0 vs. 0 patients have significant higher values of pro-thrombotic indexes; ii) non-0 vs. 0 patients have higher rate of cardiac injury; iii) non-0 vs. 0 patients have increased rate of deaths (*p* < 0.05); iv) IL-6 level is an independent predictor of cardiac injury and death; v) D-dimer is an independent predictor of death; vi) non-0 group is an independent predictor of both cardiac injury and deaths in hypertensive patients with covid-19.

In our study, non-0 patients had higher values of pro-thrombotic indexes, and higher rate of cardiac injury and death. Of interest, the AB0 blood type has been previously shown to influence the hemostasis by increasing VWF and FVIII blood levels, as well as by genetic variations and over-inflammation, that can lead to thrombosis independently of factor VIII [12]. Notably, non-0 blood group can influence the traditional risk factors for arterial or venous thrombotic events [12, 13]; besides, in patients with sepsis the non-0 blood group increases the risk for disseminated intravascular coagulopathy (DIC) independently from disease severity [12]. Equally important, endothelial dysfunction in hypertensive patients can cause cardiac injury and stroke via thromboembolism [13]. Therefore, these events could underlie the worse prognosis in patients with hypertension and covid-19. Indeed, we have shown that covid-19 infection could affect endothelial cell function leading to thrombotic complications [3]. According to the data presented here, the extent of endothelial dysfunction present in this class of patients could be further enhanced by a pro-thrombotic status in non-0patients [7]. Hence, hypertension could confer a pro-thrombotic state and over inflammation in covid-19 patients, that could be then increased in non-0 blood [2–6]. In line with this view, IL-6, a widely recognized marker of inflammation, is up regulated in non-0 vs. 0 covid-19 patients, and independently predicts cardiac injury and death. Indeed, IL-6 plays a crucial role in the cytokine release syndrome [14]. Thus, the increased IL-6 levels detected in covid-19 patients could result in worse prognosis and death [14]. Therefore, the therapeutic block of IL-6 mediated signal transduction pathway by tocilizumab, has been proposed as an effective rescue treatment in severely ill covid-19 patients [14, 15]. Hence forth, IL-6 serum levels could be used as marker of disease severity, such as predictor of worse prognosis for non-0 vs. 0 blood group of hypertensive patients with covid-19. In addition, assaying for the D-dimer could be used to add other predictive information on the risk of death in non-0 vs. 0 hypertensive patients with covid-19. Consistent with our findings, a D-dimer greater than 1 μg/mL has been proposed to help clinicians in identifying patients with poor prognosis at an early stage of covid-19 disease [16]. Indeed, augmented D-dimer level is a marker of enhanced thrombosis in patients with covid-19, [16, 17]. Thus, the functional association linking D-dimer, thrombosis, fibrinolysis and poor prognosis could be extended to hypertensive patients with non-0 blood group, identifying these patients as individuals at high risk of a severe outcome following covid-19 infection.

Finally, in hypertensive patients all these adverse events could be seen as complications of an increased inflammation, thrombosis, and fibrinolysis [18], all phenomena that are particularly enhanced in patients with non-0 blood group [19]. Indeed, AB0 blood group could cause a higher susceptibility to severe acute respiratory syndrome [19], leading to the development of neutralizing antibodies against protein-linked N-glycans [20] and acting on the stabilization of VWF [21]. Intriguingly, in a recent genomic study, authors identified a 3p21.31 gene cluster as a genetic susceptibility locus in covid-19 patients with respiratory failure [21]. Moreover, the AB0 blood-group showed a potential involvement in covid-19 disease, with a protective effect for blood group 0 as compared with the other blood groups [22].

Consequently, in the non-0 group all these adverse events could cause an increased rate of cardiac injury and death in hypertensive patients. In this regard, including elevations in serum biomarkers of cardiac damage, the standard ECG may represent a crucial test in the diagnosis of myocardial injury or heart rhythm disturbances in patients with covid-19 [23]. Indeed, ECG abnormalities, independently from the severity of pulmonary tract infection, could reflect a wide spectrum of cardiovascular complications and frequently occur after negative nasopharyngeal swabs [23]. However, ECG abnormalities of covid-19 are still undefined, particularly during the acute phase of the disease [23].

In this sense, it is critical to note that we found that the non-0 blood group results in 2.6-fold and 3.7-fold increased risk to develop cardiac injury and death in hypertensive patients with covid-19.

Our study is not exempt from limitations. For instance, we did not report data on magnetic resonance imaging or echocardiography to determine the features of myocardial injury. However, we diagnosed cardiac injury by evaluation of hs-TNI serum increase and ECG findings. Thus, we cannot have definitive data and evidence about the mechanisms of covid-19 directly heart injury. Thereby, this aspect requires further studies in order to be confirmed. Again, we did not evaluate effects of 0 vs. non-0 blood group in non-hypertensive covid-19 patients, that could be seen as control group and could limit the generalization of the present study results in overall population.

## Conclusions

Taken together, our data indicate that covid-19 associated coagulopathy should be carefully managed in hypertensive patients with non-0 group as critically ill patient because such association could result in an increased risk of unfavorable outcomes as cardiac injury and death via inflammatory and hyper-coagulative mechanisms. Therefore, we speculate that targeted anticoagulant therapies have to be introduced early in these high-risk covid-19 patients, namely hypertensive individuals with non-0 blood group, in order to reduce cardiac injury and death. Further studies conducted on larger populations are needed to confirm these results.

## Data Availability

the datasets used and/or analyzed during the current study available from the corresponding author on reasonable request.

## Abbreviations

ACE2: Angiotensin converting enzyme 2
ACEi: Angiotensin converting enzyme inhibitors
ARB: Angiotensin receptor blockers
CK-MB: Creatinine kinase–myocardial band
CT: Computed tomography
DIC: Disseminated intravascular coagulopathy
hs-TNI: High-sensitivity troponin
IL-6: Interleukin-6
OR: Odds ratios
VWF: Von Willebrand factor
